# Anticancer Potential of Xanthohumol and Isoxanthohumol Loaded into SBA-15 Mesoporous Silica Particles against B16F10 Melanoma Cells

**DOI:** 10.3390/ma15145028

**Published:** 2022-07-19

**Authors:** Tamara Krajnović, Nebojša Đ. Pantelić, Katharina Wolf, Thomas Eichhorn, Danijela Maksimović-Ivanić, Sanja Mijatović, Ludger A. Wessjohann, Goran N. Kaluđerović

**Affiliations:** 1 Institute for Biological Research “Siniša Stanković”—National Institute of Republic of Serbia, University of Belgrade, Bulevar Despota Stefana 142, 11060 Belgrade, Serbia; tamara.krajnovic@ibiss.bg.ac.rs (T.K.); nelamax@ibiss.bg.ac.rs (D.M.-I.); sanjamama@ibiss.bg.ac.rs (S.M.); 2 Department of Engineering and Natural Sciences, University of Applied Sciences Merseburg, Eberhard-Leibnitz-Straße 2, 06217 Merseburg, Germany; pantelic@agrif.bg.ac.rs (N.Đ.P.); thomas.eichhorn@hs-merseburg.de (T.E.); 3 Department of Chemistry and Biochemistry, Faculty of Agriculture, University of Belgrade, Nemanjina 6, 11080 Belgrade, Serbia; 4 Department of Bioorganic Chemistry, Leibniz Institute of Plant Biochemistry, Weinberg 3, 06120 Halle (Saale), Germany; katharina.wolf@ipb-halle.de (K.W.); wessjohann@ipb-halle.de (L.A.W.)

**Keywords:** xanthohumol, isoxanthohumol, SBA-15, cytotoxicity, melanoma

## Abstract

Xanthohumol (XN) and isoxanthohumol (IXN), prenylated flavonoids from *Humulus lupulus*, have been shown to possess antitumor/cancerprotective, antioxidant, antiinflammatory, and antiangiogenic properties. In this study, mesoporous silica (SBA-15) was loaded with different amounts of xanthohumol and isoxanthohumol and characterized by standard analytical methods. The anticancer potential of XN and IXN loaded into SBA-15 has been evaluated against malignant mouse melanoma B16F10 cells. When these cells were treated with SBA-15 containing xanthohumol, an increase of the activity correlated with a higher immobilization rate of XN was observed. Considering the amount of XN loaded into SBA-15 (calculated from TGA), an improved antitumor potential of XN was observed (IC_50_ = 10.8 ± 0.4 and 11.8 ± 0.5 µM for SBA-15|XN2 and SBA-15|XN3, respectively; vs. IC_50_ = 18.5 ± 1.5 µM for free XN). The main mechanism against tumor cells of immobilized XN includes inhibition of proliferation and autophagic cell death. The MC_50_ values for SBA-15 loaded with isoxanthohumol were over 300 µg/mL in all cases investigated.

## 1. Introduction

Cancer is a group of diseases involving abnormal cell growth that can invade or spread to other parts of the body [1,2,3]. Statistical analyzes have shown that the countries of the European Union in 2020 had almost 1.3 million deaths from cancer, which is 4.7% higher in comparison to 2015 [4]. Additionally, cancer is considered the second leading cause of death in the USA [5]. The causes of tumor occurrence and development are not entirely understood [6]. Still, it is considered that inadequate diet, low physical activity, and other global economic trends affect the appearance of tumors and other chronic diseases [7]. Usually, cancer does not show any symptoms in the early stages, while in the later phase it is often too late for successful therapeutic intervention. Apart from biotechnological and scientific improvements in medicine, chemotherapy, radiation, and surgery are still leading therapeutic approaches in cancer treatment.

**Figure 1:**
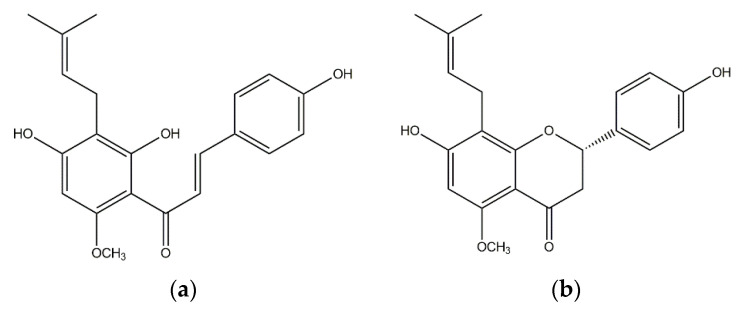
Structures of xanthohumol (**a,** XN) and isoxanthohumol (**b**, IXN).

Over the last several decades, about 500,000 natural and synthetic chemical substances have been investigated for their antiproliferative activity, but unfortunately, only a few of them are in widespread use today [8]. Some of the frequently used drugs in cancer treatment are based on cytotoxicity triggered by interacting with the DNA or microtubules. Cisplatin, discovered by Rosenberg in 1965, has had an impressive influence on cancer chemotherapy and today is the most used cure in the treatment of several types of tumors [9,10,11,12]. The application of metal-based drugs is often limited due to their severe drawbacks such as neurotoxicity, nephrotoxicity as well as ototoxicity [13,14]. Therefore, researchers put a great effort in finding potential anticancer drugs based on natural products [15,16,17,18,19,20]. It is well known that polyphenolic compounds, widespread in the plant kingdom, are archetypal antioxidants and may reduce the risk of many inflammatory (incl. cancer) and degenerative diseases [21,22,23]. Flavonoids belong to an extremely broad group of secondary plant metabolites with changeable phenolic structures, and they can be found in various fruits and vegetables [24]. Xanthohumol (XN) and isoxanthohumol (IXN), Figure 1, as representatives of prenylflavonoids, are natural products found in the female inflorescences of *Humulus lupulus*, known as hops [25,26,27]. These compounds contribute to the bitterness and taste of hops [28]. Antiproliferative activity of xanthohumol has been reported against breast (MCF-7), ovarian (A2780), prostate (PC3 and DU145), and colon (HT-29, CaCo-2, HCT115, SW620) cell lines [25,29,30]. Furthermore, XN has been shown to act as an inhibitor of human cytochrome P450 isozymes as well as inducer of quinone reductase in mouse Hepa 1c1c7 cells [31]. Xanthohumol also triggered apoptosis in 3T3-L1 adipocytes through the mitochondrial pathway and inhibited preadipocyte maturation [32]. On the other hand, the biological activity of isoxanthohumol has not been investigated in detail, but it has been found to have an estrogenic effect in breast tissue [33] and recently some of us found it to be active against metastatic melanoma and as booster of anticancer drug activity [34,35].

Lately, scientists are making great efforts to improve the delivery and effectiveness of drugs using different approaches such as loading to polylactic acid microspheres, lipid nanoparticles or modifying them by glycosylation [36,37,38,39,40]. One of the possibilities for improving delivery and targeting is the use of mesoporous silica nanoparticles such as nontoxic SBA-15 (Santa Barbara Amorphous 15) [41]. Recently, it was reported that SBA-15 loaded with cisplatin, organotin (IV) compounds or titanocene dichloride and its derivatives can reduce tumor cell growth [42,43,44,45,46].

The aim of this study is the evaluation of the in vitro anticancer potential of xanthohumol and isoxanthohumol loaded with different amounts into mesoporous silica nanoparticles SBA-15 against the B16F10 melanoma cell line using MTT and CV colorimetric assays. Therefore, the preparation and characterization of SBA-15 and SBA-15 loaded with xanthohumol and isoxanthohumol were also performed. Our data indicate that loading of XN into SBA-15, controversially to IXN, changed the anticancer potential of XN from toxic to cytostatic, resulting in a reduction of dividing potential. In addition, the apoptosis inducing property of XN is transformed into a preference of autophagic cell death, disabling tumor repopulation in response to apoptotic-induced cell proliferation, a mechanism that is tightly connected with therapy failure in advanced forms of cancer [47,48,49].

## 2. Materials and Methods

### 2.1. Materials

Pluronic 123 (P123), tetraethyl orthosilicate (TEOS), fetal calf serum (FCS), RPMI-1640, phosphate-buffered saline (PBS), l-glutamine, penicillin/streptomycin, dimethyl sulfoxide (DMSO), 3-(4,5-dimethylthiazol-2-yl)-2,5-diphenyltetrazolium bromide (MTT), crystal violet (CV), 3-methyladenine (3-MA) and carboxyfluorescein diacetate succinimidyl ester (CFSE) were purchased from Sigma (St. Louis, MO, USA). Annexin V-FITC (AnnV) was obtained from BioLegend (San Diego, CA, USA), propidium iodide (PI) from BD Pharmingen (San Diego, CA, USA) and ApoStat from R&D Systems (Minneapolis, MN, USA) while acridine orange (AO) was bought from Labo Moderna (Paris, France). Mouse skin melanoma B16F10 cell line was kindly obtained from the Leibniz Institute for Plant Biochemistry, Halle, Germany. Xanthohumol and isoxhanthohumol were purchased from Orgentis (Gatersleben, Germany,). SBA-15 was synthesized as described previously and appropriate results are given herein for comparison [50].

### 2.2. Characterization of the Mesoporous Silica Nanoparticles (MSNs)

SEM imaging was performed on VEGA3 (Tescan). Energydispersive X-ray spectroscopy (EDX) experiments were conducted on a VEGA3 microscope with an element detector from EDAX Inc. Small angle X-ray scattering (SAXS) measurements were performed on a D8 ADVANCE (Bruker) X-ray diffraction system. Autosorb iQ/ASiQwin (Quantachrome Instruments, Anton Paar, QuantaTec Inc., Boynton Beach, FL, USA) was employed for nitrogen adsorption–desorption measurements. Thermogravimetric analyses (TGA) were performed on a Netzsch TG 209 F1 Iris instrument (Netzsch Holding, Selb, Germany). Zetasizer Ultra (Malvern Analytics) was applied for DLS measurements. UV/Vis spectra were recorded with MCS621 (Carl Zeiss).

### 2.3. Preparation of SBA-15

For the synthesis of materials containing XN and IXN, SBA-15 prepared as detailed in ref. [45] was used. Yield: 15.6 g; hydrodynamic diameter: 819 nm; BET surface: 544.50 m^2^ g^−1^; pore volume: 0.97 cm^3^ g^−1^; pore diameter: 5.44 nm; wall thickness: 3.7 nm; XRD (2*θ* in °, Miller indices): 1.0034 (100), 1.6475 (111), 1.8846 (200); lattice parameter (nm): 9.2.

### 2.4. Loading of Mesoporous Silica Nanoparticles

XN or IXN were suspended in toluene (20 mL) each. To the 300 mg of activated SBA-15 (pre-dried under vacuum for 6 h at 160 °C) the suspension of XN or IXN was added, and the mixture was stirred at 60 °C for 24 h with the indicated amounts (see Table 1) of XN or IXN and SBA-15. Afterwards, the suspension was filtered, and the isolated material was washed successively with toluene (2 × 10 mL). The obtained materials were dried under vacuum at room temperature and lyophilized for 24 h. The amount of used drugs, yields, loading rates, encapsulation efficiency and physical parameters are given in Table 1.

### 2.5. Drug Release Study

2 mg of nanoparticles (SBA-15|XN3 and SBA-15|IXN3) were suspended in 1 mL of PBS (pH = 7.4) or acetate buffer (pH = 4.7) at room temperature. For drug release kinetics after 1 min, 30 min, 1, 2, 4, 6, 24, and 48 h of sample shaking, suspensions were centrifuged and supernatants were analysed using UV-Vis spectrophotometer. Standard solutions of XN and IXN were prepared by dissolving of 13.3 mg/L XN and 3 mg/L IXN, respectively, in 50 mL ethanol and 250 mL of the appropriate buffer solution.

### 2.6. Antitumor Investigation

#### 2.6.1. Cell Line

B16F10 melanoma cell line was used for determining the anticancer activities of the selected compounds. The cells were grown in T75 flasks containing 10 mL of complete medium. Nutrient medium was RPMI-1640 supplemented with 10% fetal calf serum, l-glutamine, and penicillin/streptomycin. The cells were examined daily microscopically. When the cell density reached 80% of the total flask volume, a passage was carried out. The cells were washed with 5 mL of PBS (phosphate buffered saline) and then the PBS was discarded. 1 mL of trypsin was added to the flask and the flask was incubated for 3 min at 37 °C under 5% CO_2_ in order to detach the cells. The trypsin was deactivated by adding 9 mL of complete medium to the flask, the walls of the flask were washed several times and the cell suspension was transferred to 15 mL Falcon tubes and 50 μL were transferred to an Eppendorf tube. The Falcon tube was centrifuged at 1000 rpm for 3 min, meanwhile, the cells were counted by applying 50 μL of 4% trypan blue to the 50 μL of cell suspension in the Eppendorf tube and 10 μL were transferred to a counting slide, and then inserted into the cell counter. After the centrifugation of the Falcon tube, the medium was discarded and replaced with 10 mL of complete medium. Depending on the cell count of the cell line, a specific number of cells was transferred to a new flask and the volume was completed to 10 mL using the complete medium.

#### 2.6.2. Treatment of B16F10 Cells with XN, IXN and MSNs

The surface growth area of the wells used in seeding was taken into consideration in addition to the growth ratio of the cell line. For 96-well plates, 7.000, 5.000 and 3.000 cells were seeded per well. After 24 h of seeding, the cells were treated with XN and IXN (0–100 µM) for 48 h or MSNs (0–300 µg/mL) for 24, 48 and 72 h. XN and IXN were dissolved in DMSO while stock solutions as well as working concentrations of MSNs counterparts were prepared in culture medium immediately before the treatment.

#### 2.6.3. MTT Assay

After the incubation period, the cells were washed with PBS and then the PBS was discarded. Afterwards, MTT (3-(4,5-dimethylthiazol-2-yl)-2,5-12 diphenyltetrazolium bromide) staining solution (0.5 mg/mL) was applied to each well and the cells were incubated for 30–45 min at 37 °C under 5% CO_2_. The cells were examined microscopically for formazan development. The supernatant was discarded from each well and the formazan was dissolved using DMSO [35]. The absorbance of the developed color was measured using an automated microplate reader (Spectramax from Molecular Devices) at 570 nm with a background wavelength of 670 nm. The results were presented as percentage of the values obtained from untreated cells (negative control) [43], which were arbitrarily set to 100%.

#### 2.6.4. CV Assay

At the end of cultivation period, the cells were washed with PBS, which was discarded afterwards. Then, the cells were fixed with 4% PFA (paraformaldehyde) and incubated at RT (room temperature) for 15 min. The PFA was discarded, and the cells were air-dried at RT for 5 min. Then, the cells were stained with 1% CV (crystal violet) staining solution for 15 min. The cells were washed with dd H_2_O and dried overnight at RT. After drying, the dye was dissolved in 33% acetic acid [35]. The absorbance of the developed color was measured using an automated microplate reader (Spectramax from Molecular Devices) at 570 nm with a background wavelength of 670 nm. The results were presented as percentage of the values obtained from untreated cells (negative control) [43].

The IC_50_ values, defined as 50% inhibitory concentration, were calculated with a four-parameter logistic function and presented as mean ± SD. All experiments were performed in biological triplicates.

#### 2.6.5. Wound Healing Assay

The effect of treatment with SBA-15|XN3 on cell motility in vitro was investigated by a scratch assay. B16F10 cells were seeded at 5 × 10^5^/well density and allowed to grow to 80% confluent monolayers in 6-well plates. The next day, a clean wound area in the center of all the wells was produced using a sterile pipette tip. Afterwards, the wounded cell layers were washed with fresh PBS to remove damaged cells. Cells were further incubated at 37 °C for 72 h in the presence or absence of MC_12.5_ and MC_25_ doses of SBA-15|XN3. The wounds were observed under a microscope and digitally photographed at 3 different time points (0, 48 and 72 h).

#### 2.6.6. Annexin V-FITC/PI, ApoStat and Acridine Orange Staining

B16F10 cells (2.5 × 10^5^/well) were incubated in the presence of MC_50_ dose of SBA-15|XN3 for 48 h. In order to detect potential apoptosis, cells were stained with AnnV/PI solution for 15 min at RT in the dark, according to the manufacturers’ instructions. To determine the influence of SBA-15|XN3 on caspase activity, cells were stained with ApoStat solution in PBS-5% FCS for 30 min at 37 °C, washed and resuspended in PBS. Autophagy was detected by incubating the cells with AO supervital dye in a final concentration of 10 μM in PBS at 37 °C for 15 min, washing, and resuspending in PBS. All cell samples were analyzed with CyFlow^®^ Space Partec using PartecFloMax^®^ software (Partec GmbH, Münster, Germany).

#### 2.6.7. CFSE Staining

The impact of SBA-15|XN3 on the B16F10 cell proliferation rate was measured by prestaining the cells with 1 μM CFSE in PBS-0.1% FCS for 10 min at 37 °C after which they were incubated with MC_50_ dose of SBA-15|XN3. At the end of 48 h long incubation period, cells were washed, trypsinized, resuspended in PBS and the samples were analyzed with CyFlow^®^ Space Partec using PartecFloMax^®^ software.

#### 2.6.8. Statistical Analysis

The results are presented as means ± SD of triplicate cultures from one representative of three repeated experiments with similar results unless specified otherwise. The significance of the differences between treatment and control was assessed by analysis of variance (ANOVA), followed by a Student-*t* test.

## 3. Results and Discussion

### 3.1. Immobilization of XN and IXN into SBA-15 Particles

SBA-15 is prepared by a standard sol-gel procedure and afterwards calcined [50]. Before preparation of different MSNs, SBA-15 was activated (160 °C, 6 h, in vacuum) and used for loading of various amounts of XN and IXN. Firstly, loading of XN and IXN (in ratio: SBA-15, always 300 mg; XN and IXN, 30, 60 or 90 mg) was performed at 60 °C overnight.

The white powdery products of immobilized XN and IXN into SBA-15 were characterized with SEM, nitrogen adsorption-desorption isotherms and small-angle X-ray scattering experiments (SAXS) as well as thermogravimetric analysis (TGA).

**Figure 2:**
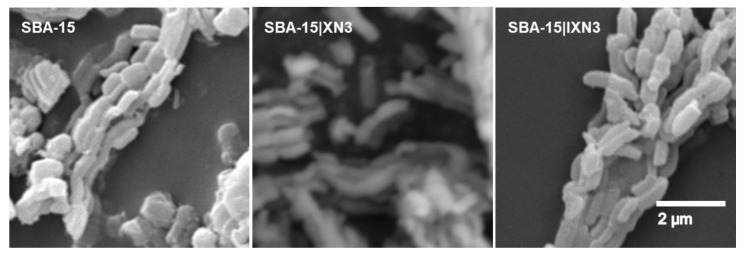
SEM images of SBA-15, SBA-15|XN3 and SBA-15|IXN3, as example.

SEM revealed the morphological properties of the synthesized materials. As can be seen in Figure 2, the SEM analysis showed that the material consists of particles which have a capsular to cylindrical shape (approx. 800 × 400 nm). The material is therefore maintained in its macroscopic shape and is stable to the used procedures.

DLS measurements (Figures S1–S7) revealed that the hydrodynamic diameters of the SBA-15 as well as immobilized XN and IXN into SBA-15 are between ca. 790–890 nm. The nitrogen adsorption-desorption analysis showed characteristics of type IV isotherms and the hysteresis loop showed type H1 behavior, which indicates the mesoporous structure of the material (IUPAC classification 1984), Figure 3a (see also Figures S8–S12) [51,52]. However, upon loading of XN or IXN into SBA-15 material, isotherms and hysteresis loop changed. The pure mesoporous SBA-15 material exhibits a hysteresis loop close to the ideal type H1 behavior, but loading and filling the 2D-channel pores with low amounts of drugs is broadening the loop and shifting it to lower p/p_0_ values together with lower overall absorbed volume of nitrogen, which proofs loading of the prenylflavonoids and the mesoporous structure of all materials. The specific surface area of the samples was measured utilizing nitrogen adsorption-desorption experiments (BET), while the mean pore diameter was calculated using the BJH (Barrett–Joyner–Halenda) method [53]. Expectedly, the specific surface area was reduced upon loading correlating with the increasing amounts of XN and IXN in SBA-15 (545 m^2^ g^−1^, SBA-15; 434 m^2^ g^−1^, SBA-15|XN3, 468 m^2^ g^−1^, SBA-15|IXN3, 426 m^2^ g^−1^). Additionally, the average pore volume (0.61 cm^3^ g^−1^) as well as the average pore diameter (43 Å) of SBA-15|XN and SBA-15|IXN decreased compared to unfunctionalized SBA-15 (0.97 cm^3^ g^−1^, 54 Å). The presented data indicate that XN and IXN have been immobilized into internal channels of the SBA-15.

**Figure 3:**
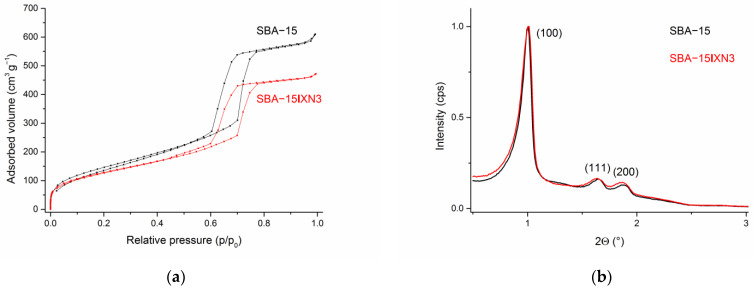
N_2_ adsorption-desorption isotherms (**a**) and SAXS pattern of SBA-15 and SBA-15|XN3 (**b**).

SAXS analysis has been used for the characterization of loaded SBA-15 materials and the corresponding patterns are shown in Figure 3b (see also Figures S13–S17). The SAXS patterns show three peaks for the pure starting material, SBA-15, 1.0034° (100), high intensity, 1.6475° (110) and 1.8846° (200), which can be indexed on the 2D hexagonal lattice with the lattice parameter values between 88 and 94 Å [50]. Small shifts were noticed for immobilized MSNs as a consequence to the loading of XN or IXN. A substantial decrease in the intensity of the diffraction peaks was noticed upon loading of XN and IXN due to a blocking of the mesopores and shrinkage in inward pore dimensions by the organic molecules. Therefore, some probes exhibited just two reflection peaks [50,54].

The loading of all substances studied was determined by thermogravimetric analysis (TGA). Loaded and unloaded SBA-15 was heated to 800 °C and mass changes equivalent to the adsorbed drug amounts were noted. The water content was corrected by comparison to pure SBA-15. A low loading rate was found for all materials and therefore low encapsulation efficiency was also observed (from 8% up to 17% for SBA-15|XN3). However, higher amounts of XN or IXN used in the immobilization process resulted in a higher appropriate drug content inside SBA-15. Up to 5.0% of XN and 3.5% of IXN, SBA-15|XN3 and SBA-15|IXN, respectively, were incorporated into SBA-15 (Figures S18–S24).

A drug release study was followed for the SBA-15|XN3 (as the most active nanomaterials, vide infra) and SBA-15|IXN3 using UV-Vis spectrophotometry (Figure S25). The release rates of XN and IXN from the SBA-15|XN3 and SBA-15|IXN3, respectively, were studied in PBS (pH = 7.4) and acetate buffer (pH = 4.7) at room temperature. It can be noticed that the release of XN and IXN occurs very fast and after 6 h the maximum concentration is reached (Figure S25), which corresponds to the solubility of XN and IXN under the chosen conditions. Furthermore, results showed that the release rate was pH-independent. Moreover, pH has a minor effect on the released amount of drug and more XN/IXN is released with higher pH. It should be noted that at very basic or acidic pH XN forms IXN and vice versa [55].

### 3.2. In Vitro Studies

The anticancer potential of XN and IXN loaded into SBA-15, as well as free XN and IXN used as controls, was evaluated using MTT and CV colorimetric assays on B16F10 melanoma cell line after 48 h of incubation. The in vitro cytotoxic activity results are expressed as IC_50_ values [µM] for XN and IXN, and MC_50_ values [µg/mL] for loaded MSNs and presented in Table 2.

As seen in Table 2 and Figure S26, XN and IXN reduced the number of viable cancer cells in a dose-dependent manner with emphasis on XN showing better cytotoxic activity in both colorimetric tests used. The MTT assay is based on MTT tetrazolium salt reduction to formazan and relies on the mitochondria of living cells. However, this test excludes the possibility that cells often change the respiration intensity in response to different stimuli. On the other hand, the CV assay is based on staining of adherent cells with crystal violet dye, which binds to proteins and DNA, and therefore the CV test is considered as more reliable than MTT [56]. Based on that, it is noticed that the cytotoxicity of XN is approximately 3 times higher than that of IXN (Table 2, CV assay). Furthermore, loading of the XN into nanoparticles SBA-15 preserved and even potentiated its activity.

**Figure 4:**
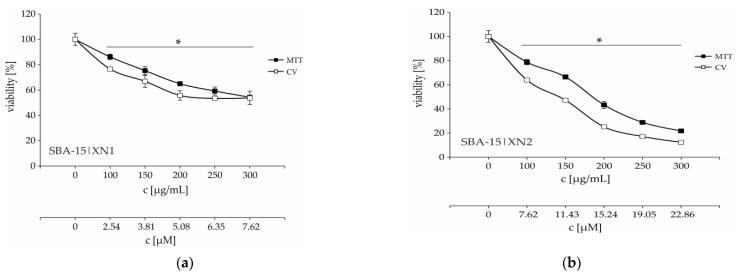

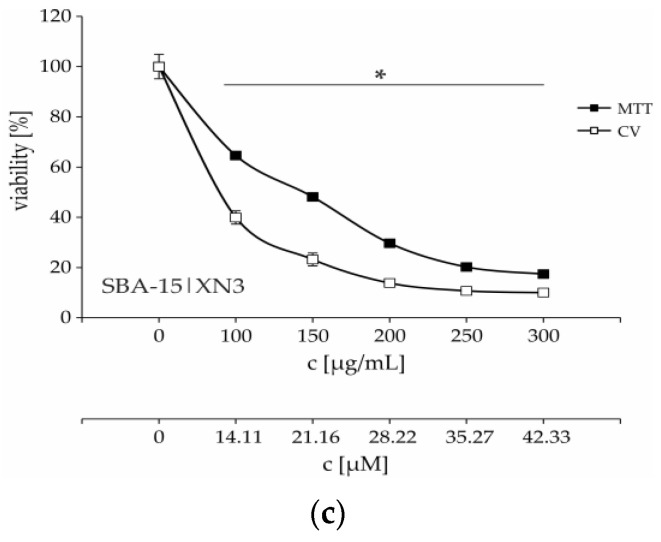
Dose-dependent viability decrease of B16F10 melanoma cells treated with various concentrations of SBA-15|XN1–SBA-15|XN3 (**a**–**c**) for 48 h. * *p* < 0.05 compared to the untreated control cells. c [μM] is calculated according to amount of XN by TGA.

According to MC_50_ values there is a trend of increasing anticancer activity with an increasing amount of XN into SBA-15 (Table 2 and Figure 4). On the other hand, according to calculated IC_50_ from MC_50_ concentrations (based on TGA), there is no significant difference between SBA-15|XN3 and SBA-15|XN2 (11.8 ± 0.5 and 10.8 ± 0.5 μM, respectively), probably due to precipitation of the substance within the nanomaterial and the establishment of an equilibrium between solution and precipitate. The solubility of the flavonoids in water or buffer is low. Nevertheless, by loading of IXN into SBA-15 (SBA-15|IXN1-SBA-15|IXN3), the MC_50_ values were not reached (>300 μg/mL). It has been previously demonstrated that SBA-15 is inactive toward cancer cell lines [43,44].

**Figure 5:**
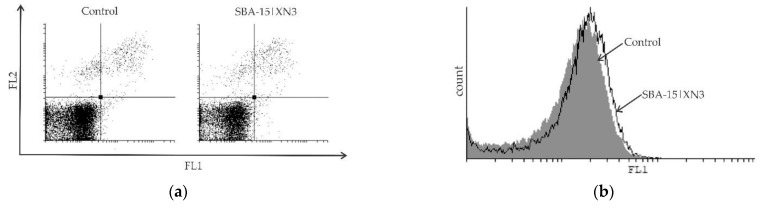

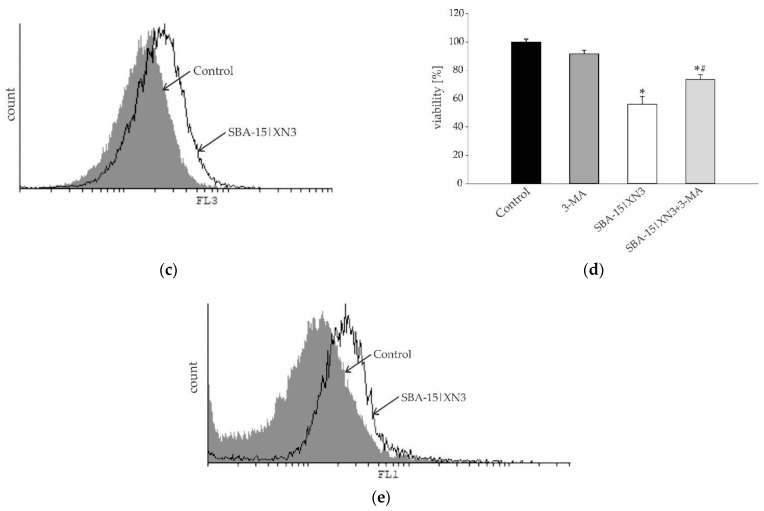
Inhibition of cellular proliferation and promotion of autophagy without induction of caspase-dependent apoptosis in B16F10 melanoma cell culture determined using Ann/PI (**a**), ApoStat (**b**), AO (**c**) and CFSE (**e**) staining, all performed after 48 h of treatment with SBA-15|XN3 and subsequently analyzed by flow cytometry. Dot plots and histograms are representative ones selected from three repeated experiments. The induction of autophagic cell death exposed to MC_50_ dose SBA-15|XN3 and 3-MA (in final concentration of 1 mM) for 48 h (**d**). Cell viability was determined by CV assay and expressed as a percentage of control values (untreated cells). The data are presented as mean ± SD obtained from three independent experiments. * *p* < 0.05 refers to untreated cultures; # *p* < 0.05 refers to SBA-15|XN3 treated cultures.

Time-resolved kinetics showed a continuous decrease of cell viability until 48 h (Figure S27, Figure 4), while an additional 24 h of cultivation led to a plateau-effect. These data indicate that the pivotal mode of action of SBA-15|XN3 is inhibition of proliferation rather than cell death. The mentioned hypothesis is further proved by cytofluorimetric analysis where apoptosis, as well as caspase activation were not found in treated cultures, while an intensified presence of autophagic vesicles and a strong inhibition of proliferation were noted after the treatment (Figure 5). Exposure of the cells to 3-methyladenine, a specific autophagy inhibitor, in parallel with an experimental drug revealed that the autophagic process partly contributed to the viability decrease. On the other hand, as previously described, XN alone can induce caspase-dependent apoptosis (Figure S28). In parallel, inhibition of proliferation was followed by abolished cell migration, as discovered by the wound healing test (Figure S29). Namely, while untreated cells filled the scratched area within 72 h, cells treated with SBA-15|XN3 in doses that decreased cell viability by 25% or 12.5% significantly affected their ability to invade the empty area. Taken together, it can be concluded that loading of XN into SBA-15 converts its activity from previously observed predominantly cytotoxic toward cytostatic behavior.

## 4. Conclusions

The natural compounds XN and IXN were immobilized into mesoporous silica SBA-15. Novel MSNs were characterized by SEM, N_2_ sorption, SAXS and TGA analysis. The prenylflavonoids were loaded up to 5.0% (XN) or 3.5% (IXN) w/w into SBA-15. SBA-15 containing XN showed a loading rate—activity dependence against B16F10 melanoma cells. Importantly, immobilization of XN into nanoparticles of SBA-15 preserved and even potentiated its activity. The antitumor potential of SBA-15|XN is based on the inhibition of cell proliferation and autophagic cell death, contrasting the previously observed apoptotic-inducing properties of free XN. This indicates that the loading of XN into SBA-15 carrier resulted not only in a quantitative but as well as in a qualitative change of its antitumor action.

## Figures and Tables

**Table 1:** SBA-15 nanomaterials.

MSN	SBA-15|XN1	SBA-15|XN2	SBA-15|XN3	SBA-15|IXN1	SBA-15|IXN2	SBA-15|IXN3
**Drug amount [mg]**	30	60	90	30	60	90
**Yield [mg]**	279	274	306	230	198	275
**Loading [%]**	0.9	2.7	5	1.1	2.3	3.5
**Encaps. efficiency [%]**	8	12	17	8	8	11
**Hydrodynamic *r* [nm]**	877	889	785	831	731	852
**BET surface [m^2^ g^−1^]**	419.99	525.11	468.26	403.92	433.1	426.39
***V*_pore_ [cm^3^ g^−1^]**	0.5	0.77	0.72	0.49	0.45	0.51
***d*_pore_ [nm]**	3.7	5.9	5.8	3.7	3.7	3.7
**Wall thickness [nm]**	5.3	3.3	3.3	4.4	4.5	5.4
**Miller ind. 2*θ* [°]**	1.1034 (100) 1.4567 (111) 2.0682 (200)	1.0035 (100) 1.6496 (111) 1.8809 (200)	1.0083 (100) 1.6339 (111) 1.8721 (200)	1.1261 (100) 2.1071 (200)	1.1193 (100) 1.6496 (111) 2.0734 (200)	1.1037 (100) 1.4191 (111) 2.0836 (200)
**Lattice param. [nm]**	9	9.2	9.2	8.1	8.2	9.1

**Table 2:** IC_50_ (μM) and MC_50_ (μg/mL) values of free and immobilized XN and IXN into SBA-15 after 48 h of treatment.

	IC_50_ (μM)		MC_50_ (μg/mL)			
Assay	XN	IXN	SBA-15|XN1	SBA-15|XN2	SBA-15|XN3	SBA-15|IXN1-SBA-15|IXN3
MTT	6.3 ± 0.5	30.3 ± 2.0	>300	189.2 ± 5.0	149.7 ± 4.7	>300
CV	18.5 ± 1.5	57.7 ± 3.6	>300	141.6 ± 4.6	83.2 ± 3.9	>300

## Data Availability

Data Supporting obtained results can be obtained from the authors upon request.

## Supplementary Materials

The following supporting information can be downloaded at: https://www.mdpi.com/article/10.3390/ma15145028/s1.
