# Supplementary material for: Anticancer Potential of Xanthohumol and Isoxanthohumol Loaded into SBA-15 Mesoporous Silica Particles against B16F10 Melanoma Cells
Source: Materials (Basel). 2022 Jul 19;15(14):5028. doi: 10.3390/ma15145028 (PMC9320346; doi:10.3390/ma15145028)
Supplement: Supplementary file 1 [file materials-15-05028-s001.zip › materials-1711859-supplementary.pdf]

Supplementary Materials

Anticancer potential of xanthohumol and isoxanthohumol loaded into SBA-15 mesoporous silica particles against B16F10 melanoma cells

Tamara Krajnović, Nebojša Đ. Pantelić, Katharina Wolf, Thomas Eichhorn, Danijela Maksimović-Ivanić, Sanja Mijatović, Ludger A. Wessjohann and Goran N. Kaluđerović

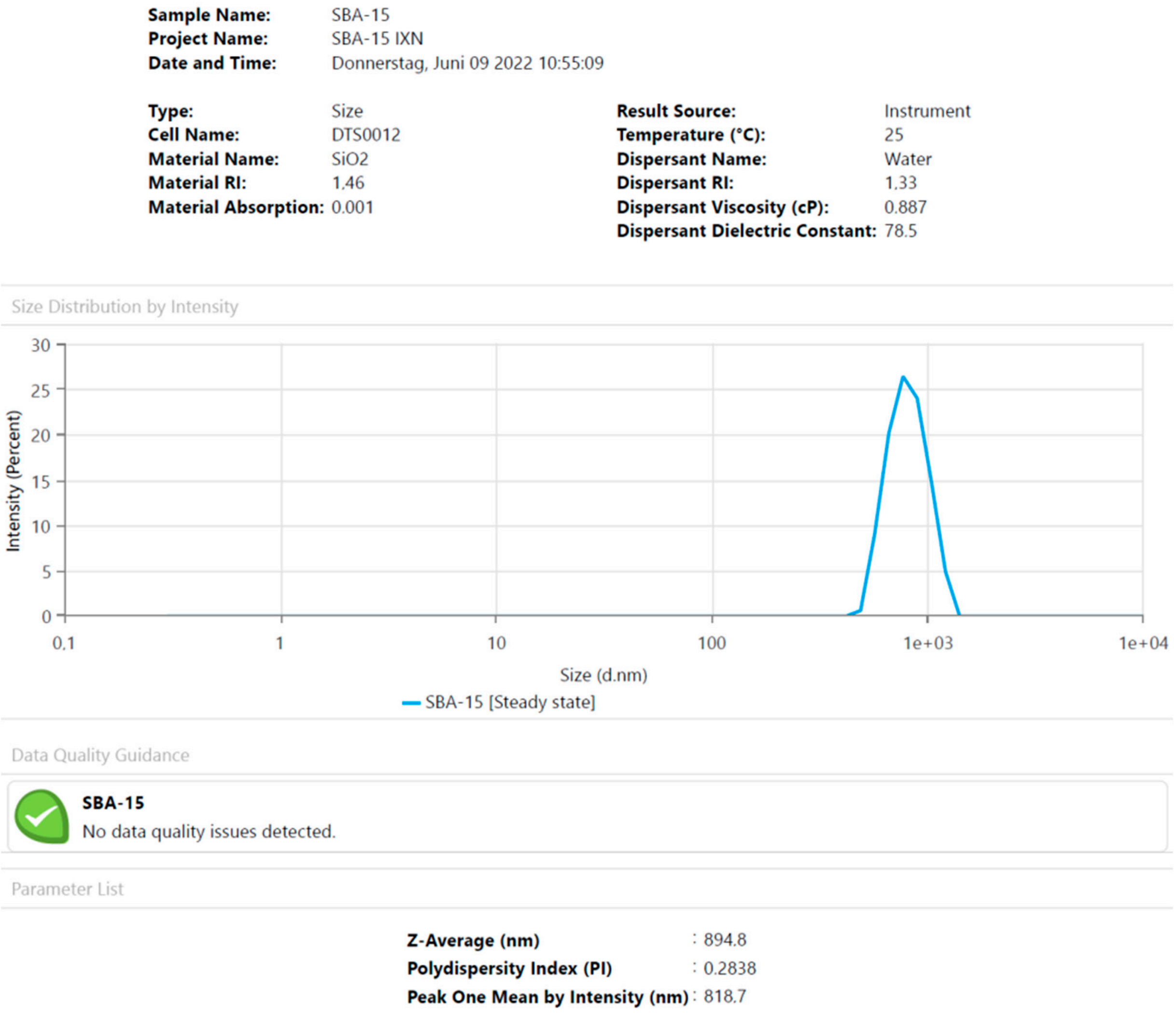

Figure S1. DLS of SBA-15.

**Sample Name:**	SBA-15 I XN1		
**Project Name:**	SBA-15 IXN		
**Date and Time:**	Donnerstag, Juni 09 2022 11:12:08		
**Type:**	Size	**Result Source:**	Instrument
**Cell Name:**	DTS0012	**Temperature (°C):**	25
**Material Name:**	SiO2	**Dispersant Name:**	Water
**Material RI:**	1.46	**Dispersant RI:**	1.33
**Material Absorption:**	0.001	**Dispersant Viscosity (cP):**	0.887
		**Dispersant Dielectric Constant:**	78.5

Size Distribution by Intensity

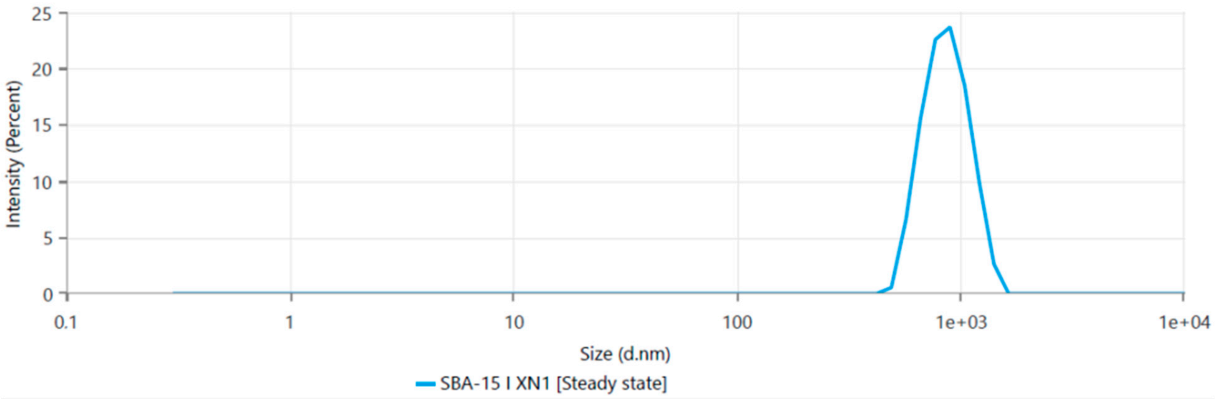

Data Quality Guidance

SBA-15 I XN1
No data quality issues detected.

Parameter List

**Z-Average (nm)**	: 1024
**Polydispersity Index (PI)**	: 0.3358
**Peak One Mean by Intensity (nm)**	: 876.8

Figure S2. DLS of SBA-15 | XN1.

**Sample Name:**	SBA-15 I XN2		
**Project Name:**	SBA-15 IXN		
**Date and Time:**	Donnerstag, Juni 09 2022 11:42:24		
**Type:**	Size	**Result Source:**	Instrument
**Cell Name:**	DTS0012	**Temperature (°C):**	25
**Material Name:**	SiO2	**Dispersant Name:**	Water
**Material RI:**	1.46	**Dispersant RI:**	1.33
**Material Absorption:**	0.001	**Dispersant Viscosity (cP):**	0.887
		**Dispersant Dielectric Constant:**	78.5

Size Distribution by Intensity

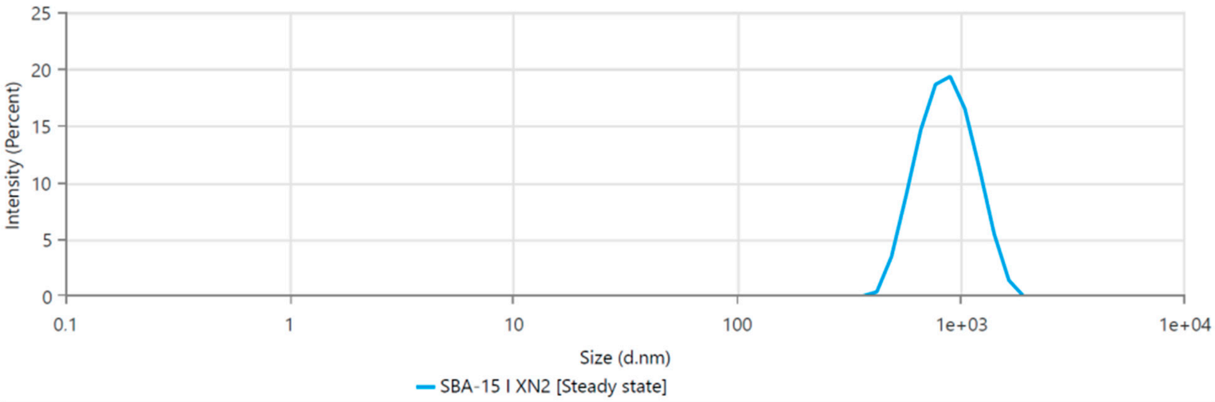

Data Quality Guidance

SBA-15 I XN2
No data quality issues detected.

Parameter List

**Z-Average (nm)**	: 957.3
**Polydispersity Index (PI)**	: 0.3159
**Peak One Mean by Intensity (nm)**	: 889.4

Figure S3. DLS of SBA-15|XN2.

Sample Name: SBA-15 I XN3
Project Name: SBA-15 IXN
Date and Time: Donnerstag, Juni 09 2022 12:17:02

**Type:**	Size	**Result Source:**	Instrument
**Cell Name:**	DTS0012	**Temperature (°C):**	25
**Material Name:**	SiO2	**Dispersant Name:**	Water
**Material RI:**	1.46	**Dispersant RI:**	1.33
**Material Absorption:**	0.001	**Dispersant Viscosity (cP):**	0.887
		**Dispersant Dielectric Constant:**	78.5

Size Distribution by Intensity

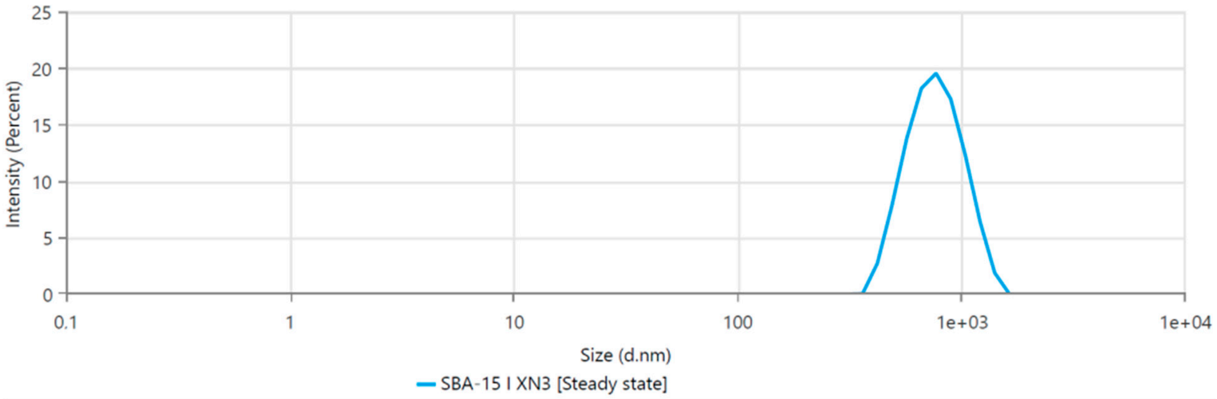

Data Quality Guidance

SBA-15 I XN3
No data quality issues detected.

Parameter List

**Z-Average (nm)**	: 790.9
**Polydispersity Index (PI)**	: 0.3955
**Peak One Mean by Intensity (nm)**	: 784.8

Figure S4. DLS of SBA-15|XN3.

**Sample Name:**	SBA-15 I IXN1
**Project Name:**	SBA-15 IXN
**Date and Time:**	Donnerstag, Juni 09 2022 12:26:22

**Type:**	Size	**Result Source:**	Instrument
**Cell Name:**	DTS0012	**Temperature (°C):**	25
**Material Name:**	SiO2	**Dispersant Name:**	Water
**Material RI:**	1.46	**Dispersant RI:**	1.33
**Material Absorption:**	0.001	**Dispersant Viscosity (cP):**	0.887
		**Dispersant Dielectric Constant:**	78.5

Size Distribution by Intensity

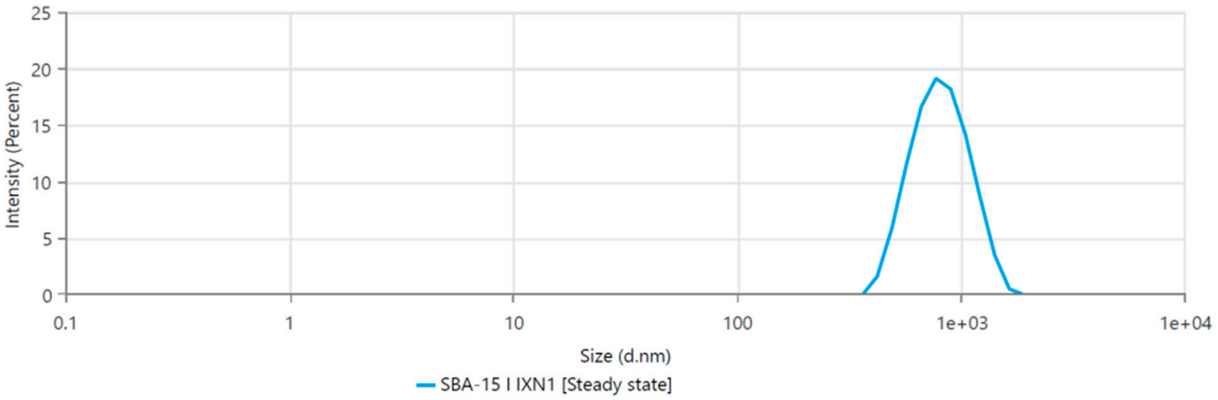

Data Quality Guidance

SBA-15 I IXN1
No data quality issues detected.

Parameter List

**Z-Average (nm)**	: 828.3
**Polydispersity Index (PI)**	: 0.3182
**Peak One Mean by Intensity (nm)**	: 831.3

Figure S5. DLS of SBA-15 I IXN1.

**Sample Name:**	SBA-15 I IXN2		
**Project Name:**	SBA-15 IXN		
**Date and Time:**	Donnerstag, Juni 09 2022 12:37:17		
**Type:**	Size	**Result Source:**	Instrument
**Cell Name:**	DTS0012	**Temperature (°C):**	25
**Material Name:**	SiO2	**Dispersant Name:**	Water
**Material RI:**	1.46	**Dispersant RI:**	1.33
**Material Absorption:**	0.001	**Dispersant Viscosity (cP):**	0.887
		**Dispersant Dielectric Constant:**	78.5

Size Distribution by Intensity

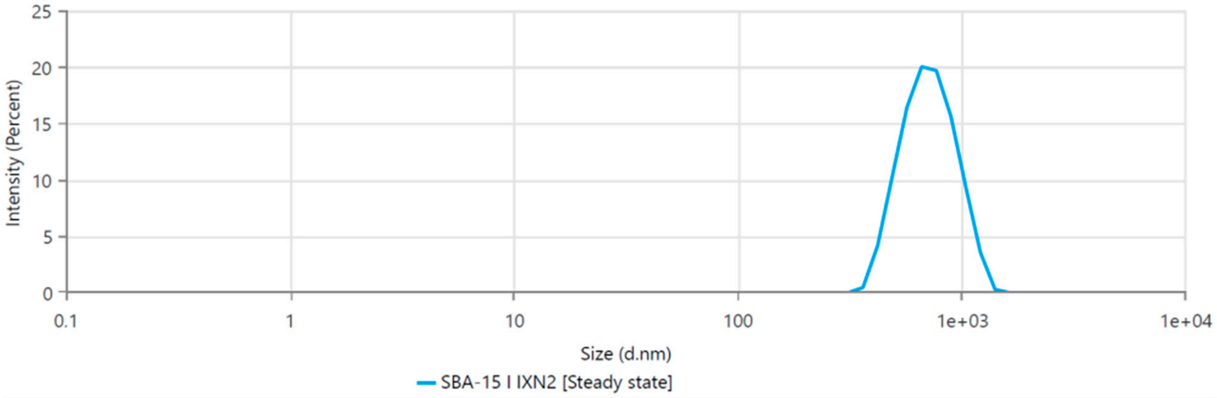

Data Quality Guidance

SBA-15 I IXN2
No data quality issues detected.

Parameter List

**Z-Average (nm)**	: 741.6
**Polydispersity Index (PI)**	: 0.3981
**Peak One Mean by Intensity (nm)**	: 731.2

Figure S6. DLS of SBA-15|IXN2.

**Sample Name:**	SBA-15 I IXN3
**Project Name:**	SBA-15 IXN
**Date and Time:**	Donnerstag, Juni 09 2022 01:02:16

**Type:**	Size	**Result Source:**	Instrument
**Cell Name:**	DTS0012	**Temperature (°C):**	25
**Material Name:**	SiO2	**Dispersant Name:**	Water
**Material RI:**	1.46	**Dispersant RI:**	1.33
**Material Absorption:**	0.001	**Dispersant Viscosity (cP):**	0.887
		**Dispersant Dielectric Constant:**	78.5

Size Distribution by Intensity

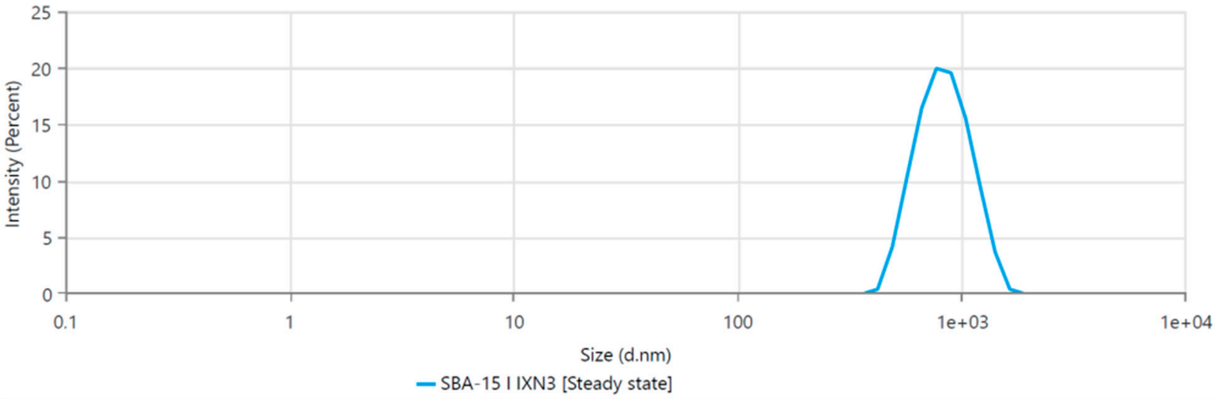

Data Quality Guidance

SBA-15 I IXN3
No data quality issues detected.

Parameter List

**Z-Average (nm)**	: 758.1
**Polydispersity Index (PI)**	: 0.3414
**Peak One Mean by Intensity (nm)**	: 852.2

Figure S7. DLS of SBA-15 I IXN3.

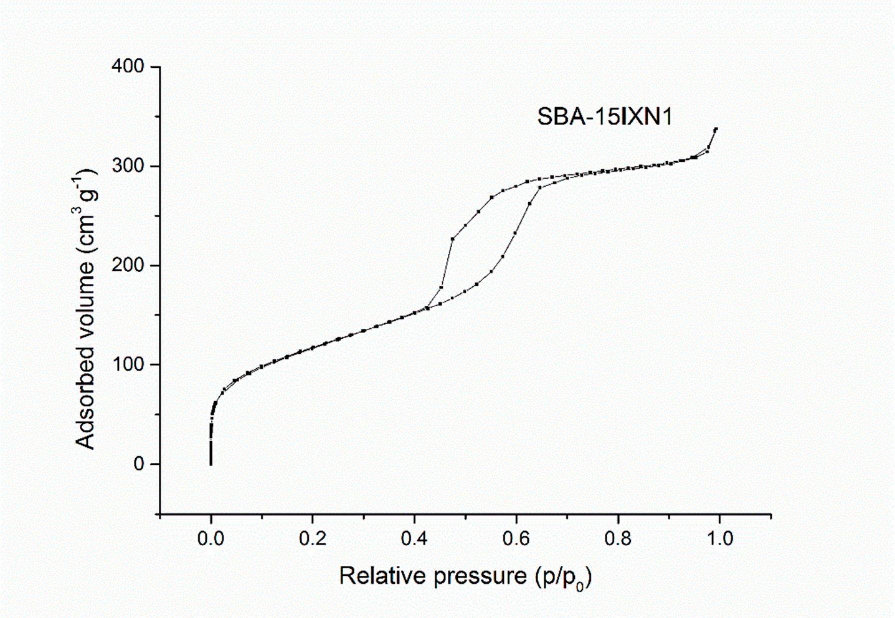

Figure S8. N_2 adsorption-desorption isotherms: SBA-15|XN1.

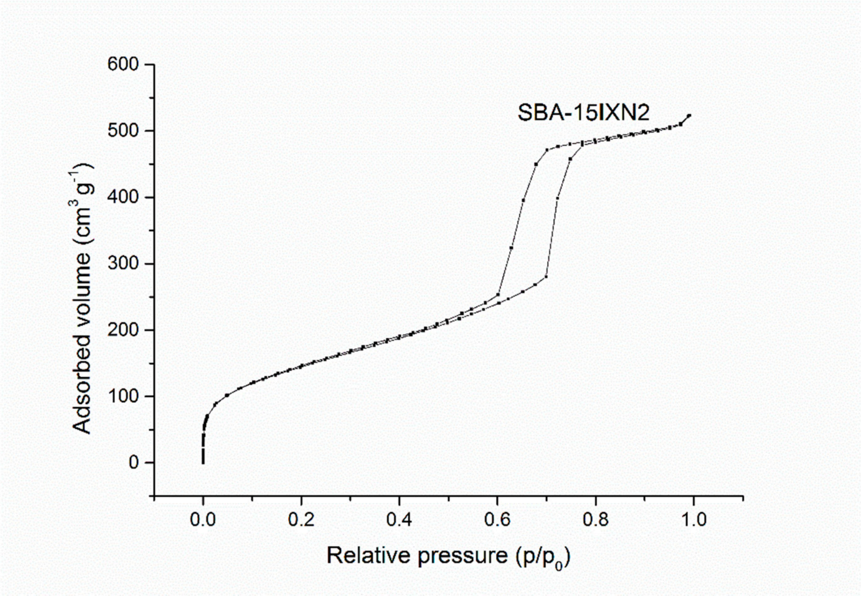

Figure S9. N_2 adsorption-desorption isotherms: SBA-15|XN2.

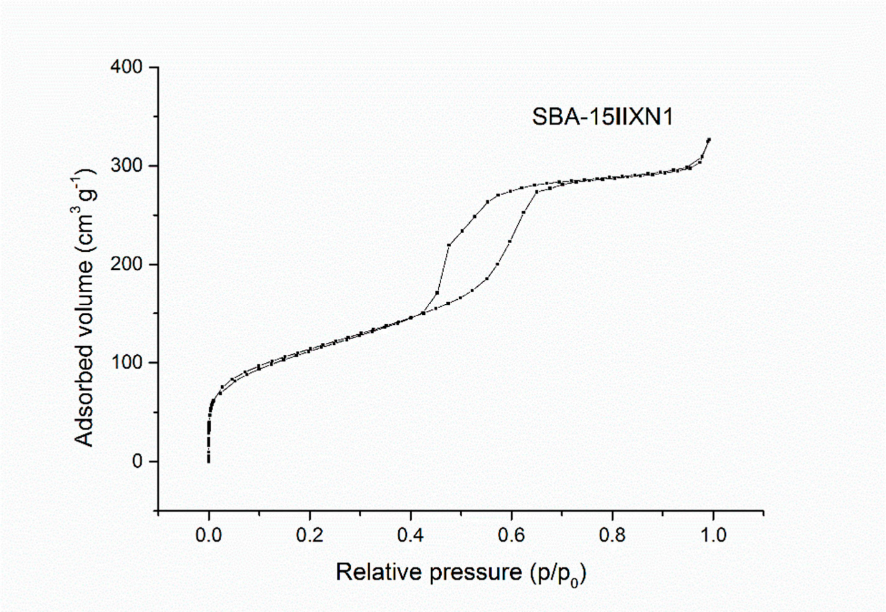

Figure S10. N_2 adsorption-desorption isotherms: SBA-15|IXN1.

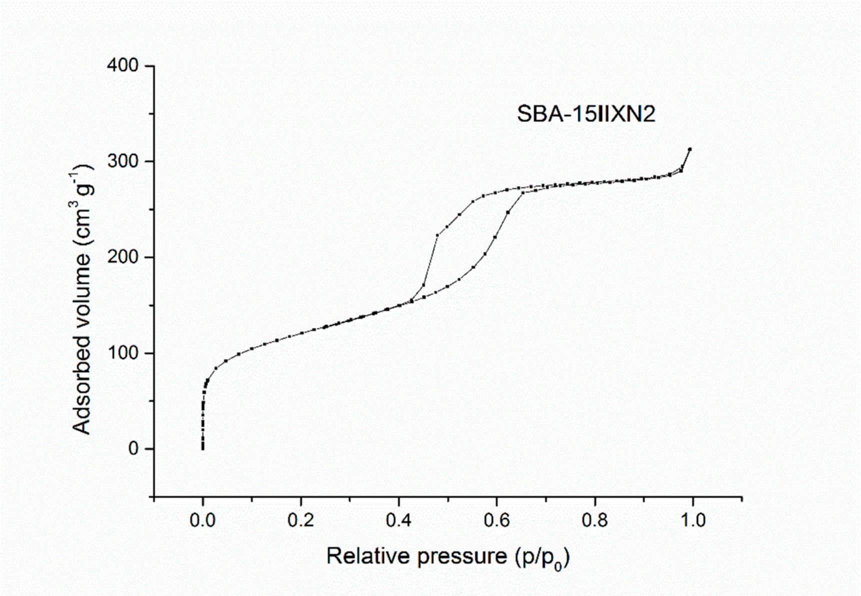

Figure S11. N_2 adsorption-desorption isotherms: SBA-15|IXN2.

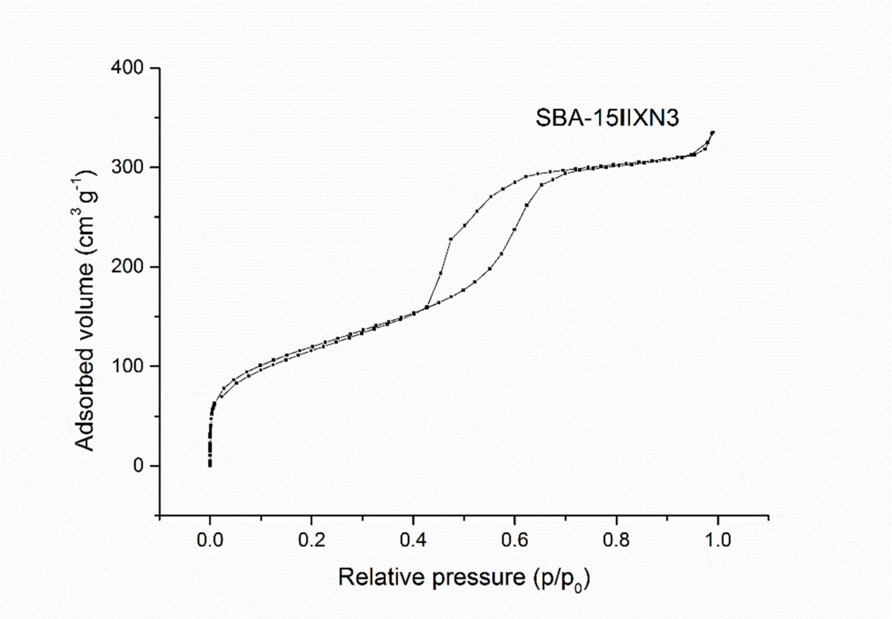

Figure S12. N_2 adsorption-desorption isotherms: SBA-15|IXN3.

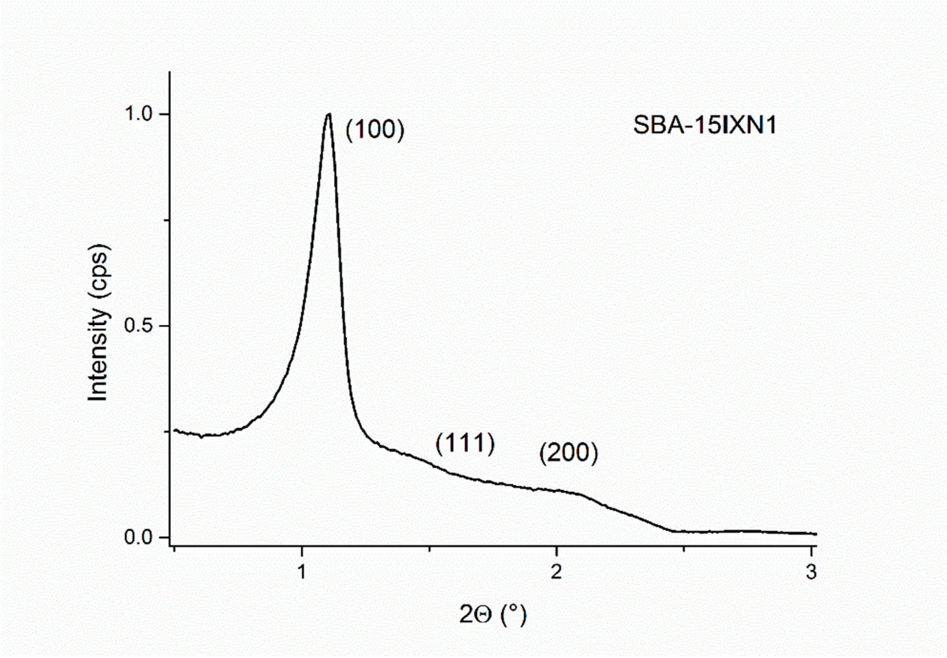

Figure S13. Small-angle X-ray scattering – SAXS: SBA-15|XN1.

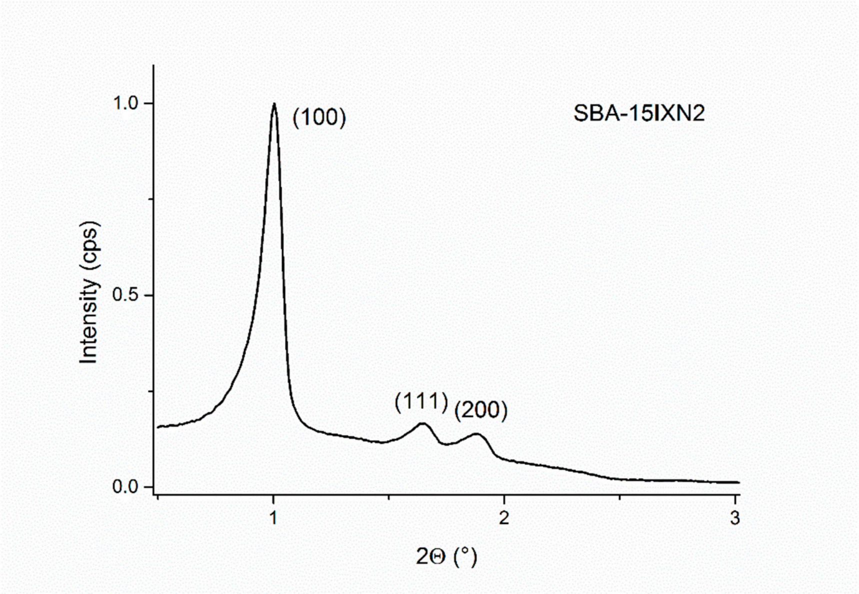

Figure S14. Small-angle X-ray scattering – SAXS: SBA-15|XN2.

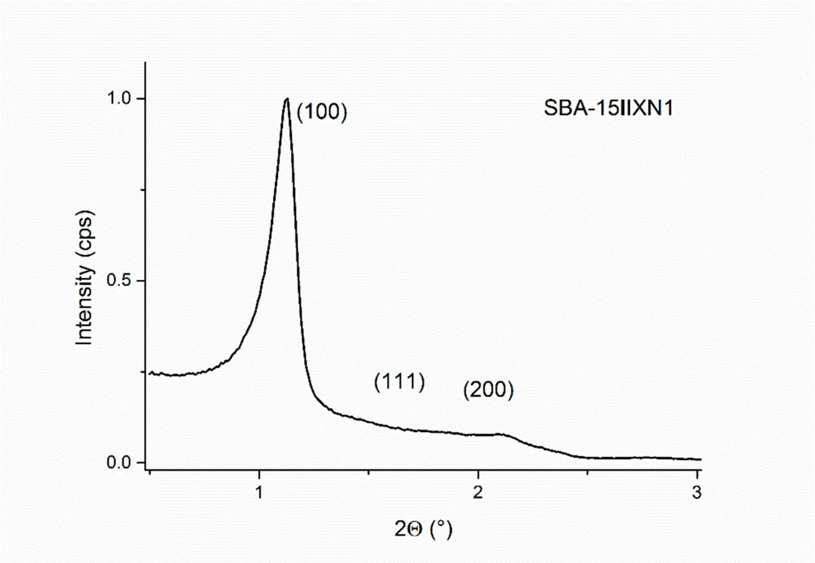

Figure S15. Small-angle X-ray scattering – SAXS: SBA-15|IXN1.

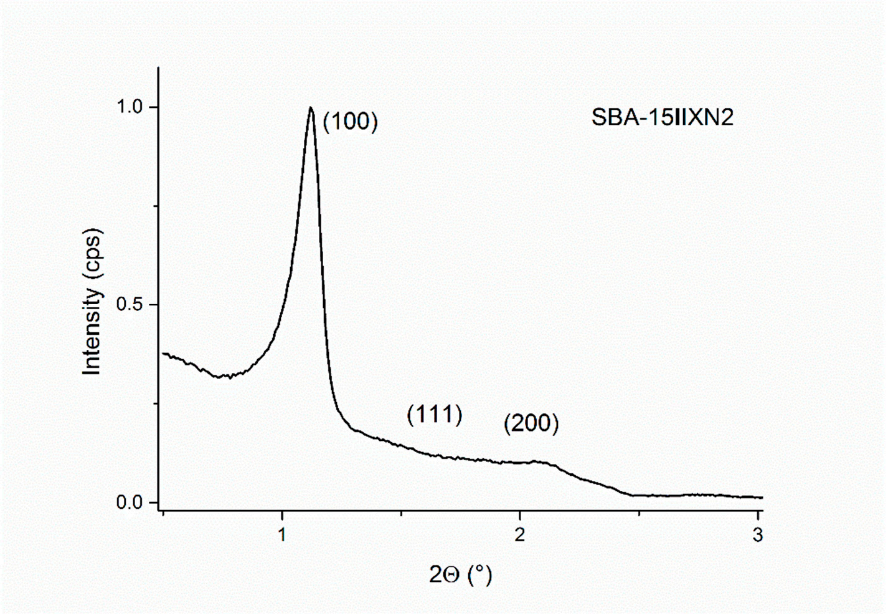

Figure S16. Small-angle X-ray scattering – SAXS: SBA-15|IXN2.

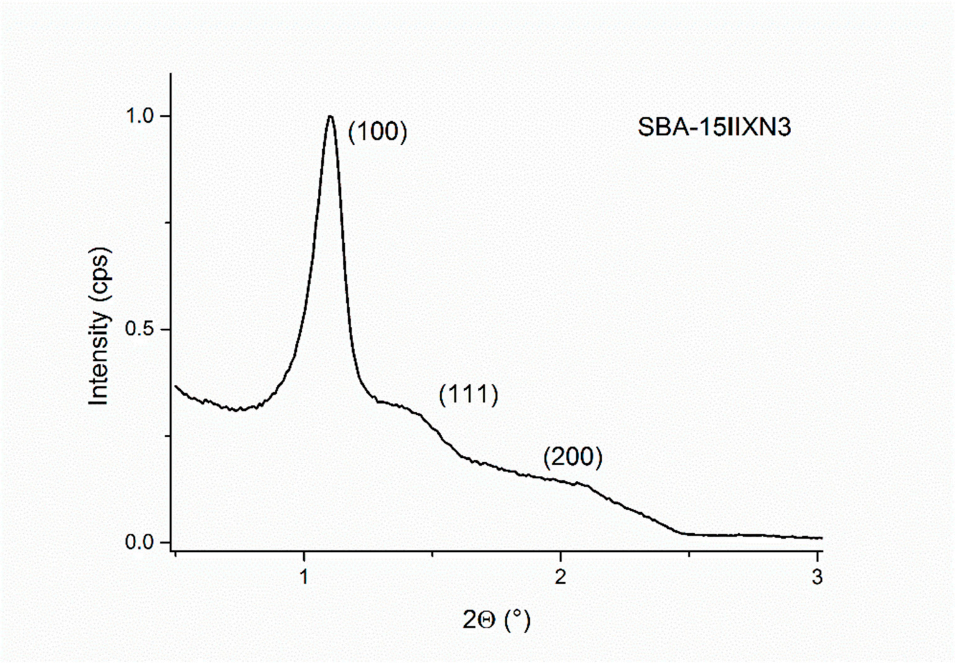

Figure S17. Small-angle X-ray scattering – SAXS: SBA-15|IXN3.

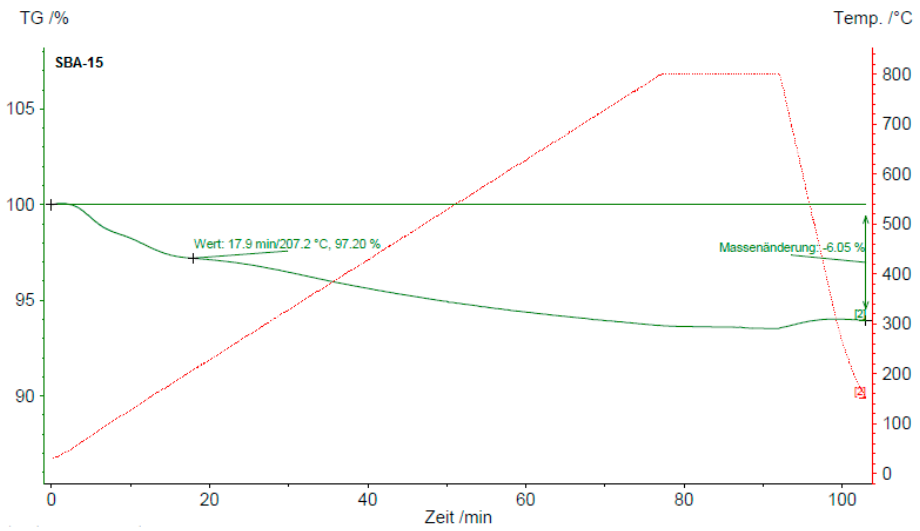

Figure S18. TGA of SBA-15.

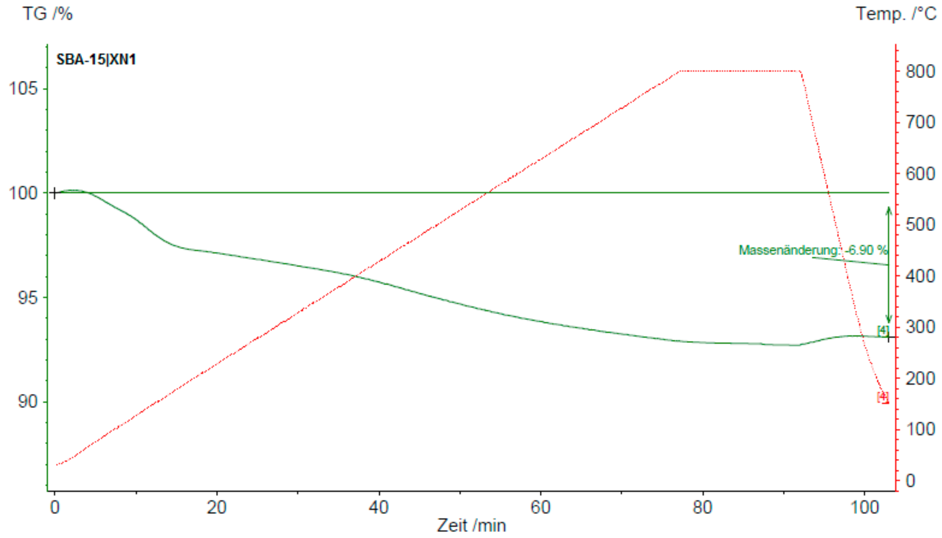

Figure S19. TGA of SBA-15|XN1.

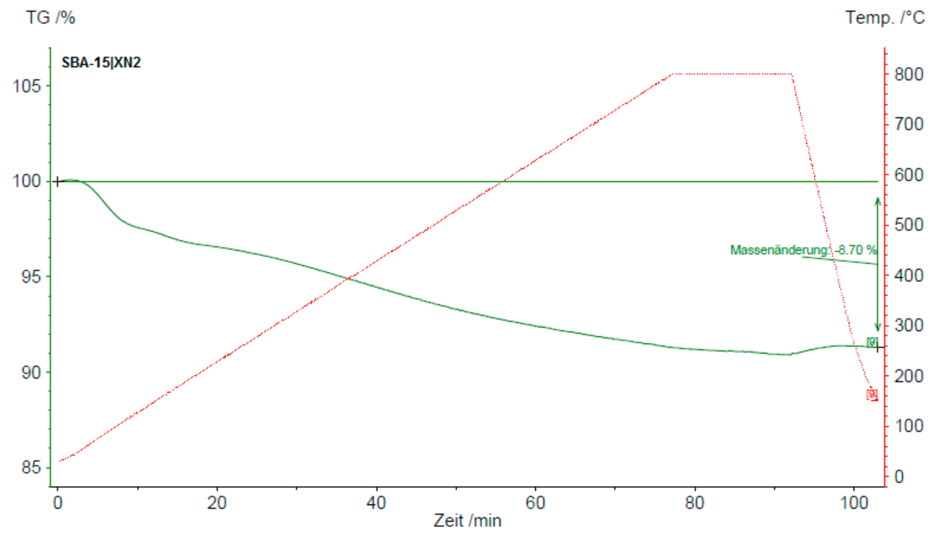

Figure S20. TGA of SBA-15|XN2.

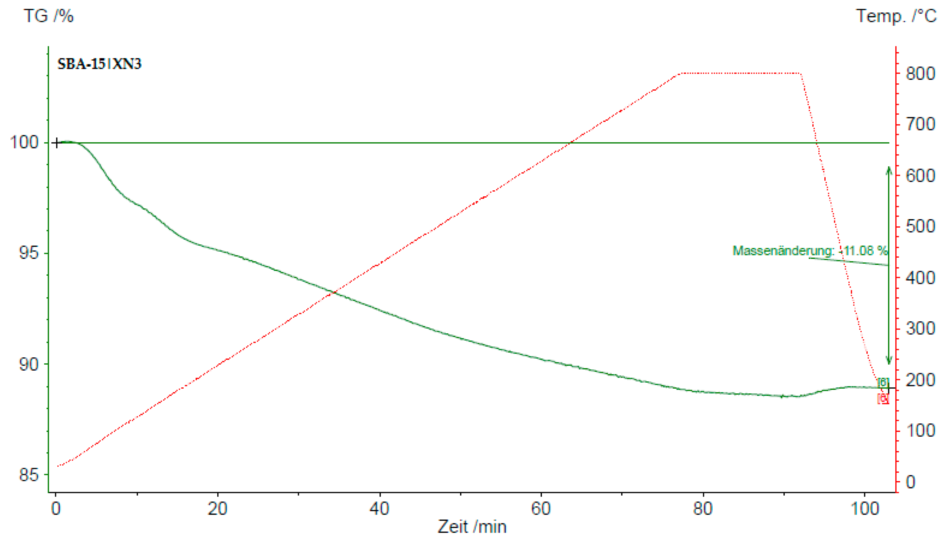

Figure S21. TGA of SBA-15|XN3.

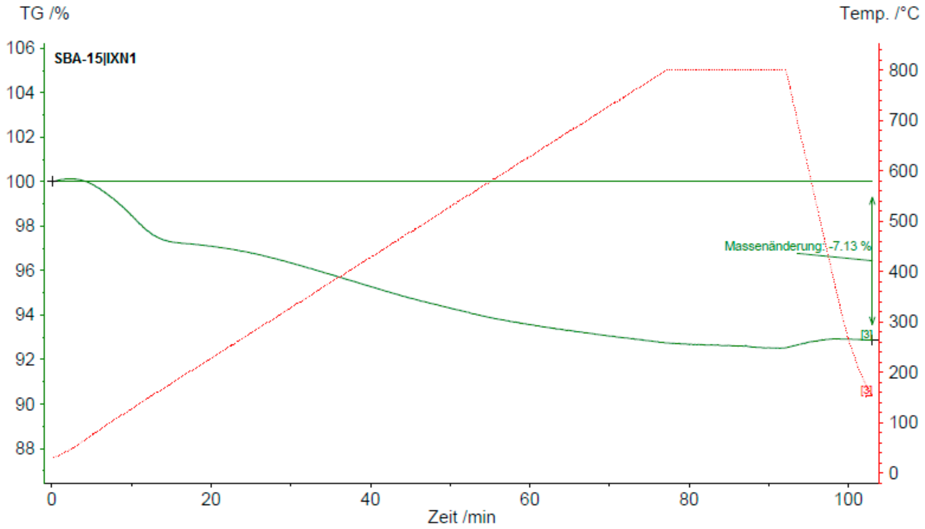

Figure S22. TGA of SBA-15|IXN1.

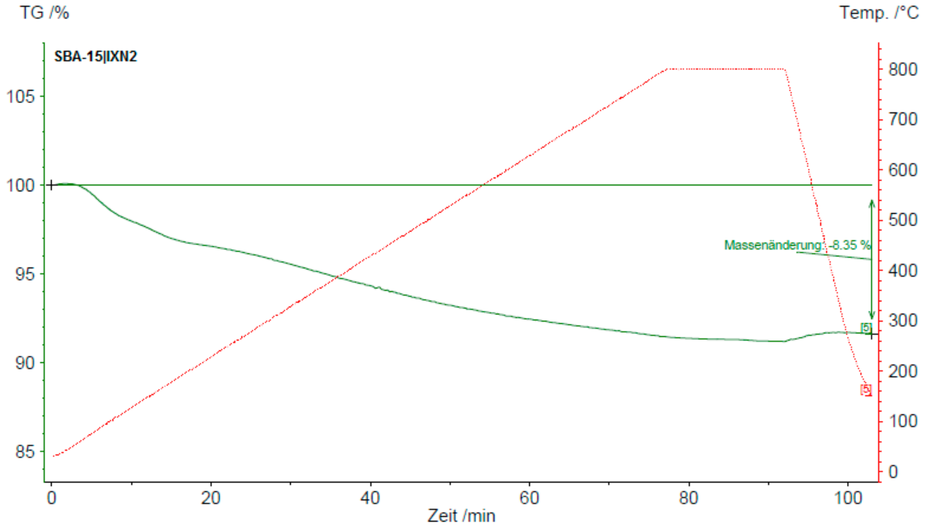

Figure S23. TGA of SBA-15|IXN2.

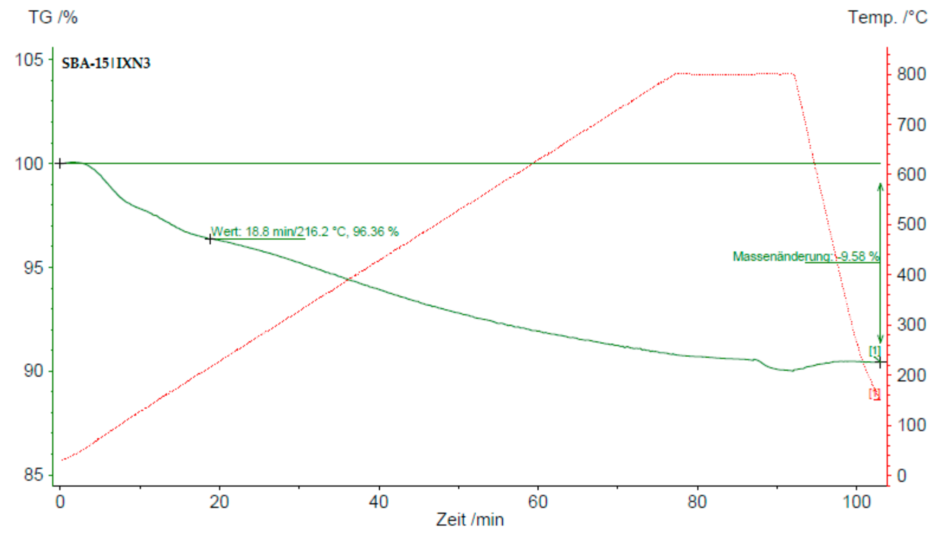

Figure S24. TGA of SBA-15 I IXN3.

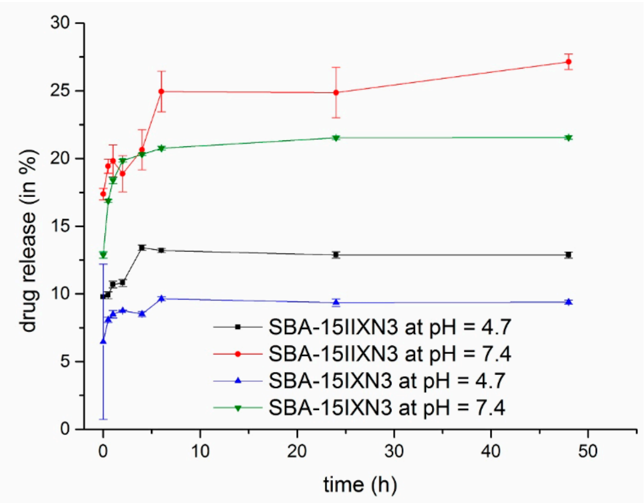

Figure S25. Release kinetics of SBA-15 I XN3 and SBA-15 I IXN3 in PBS and acetate buffer (pH = 7.4 and 4.7, respectively). Results are presented as mean ± SD from three independent experiments.

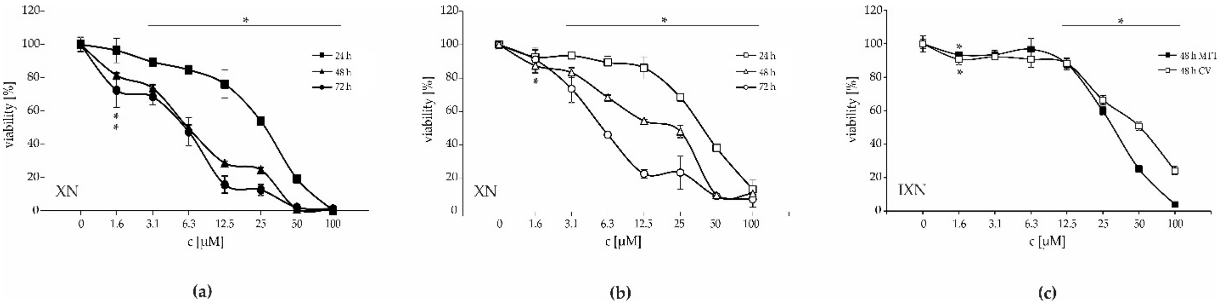

Figure S26. Dose-dependent viability decrease of B16F10 melanoma cells treated with various concentrations (1.6–100 μM) of XN (**a**, MTT; **b**, CV) and IXN (**c**). The cell viability is expressed as a percentage of control values (untreated cells), and the data are presented as mean ± SD from three independent experiments. * $p < 0.05$ compared to the untreated control cells.

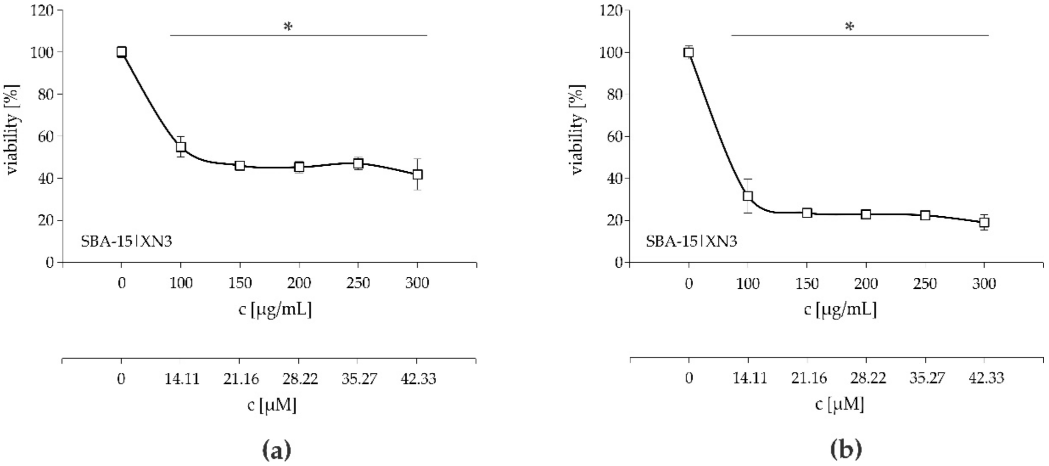

Figure S27. Viability of B16F10 melanoma cells treated with various concentrations (100–300 μg/mL) of SBA-15|XN3 for 24 h (**a**) and 72 h (**b**) determined by CV assay. The cell viability is expressed as a percentage of control values (untreated cells), and the data are presented as mean ± SD from three independent experiments. * $p < 0.05$ compared to the untreated control cells. c [μM] is calculated according to amount of XN by TGA.

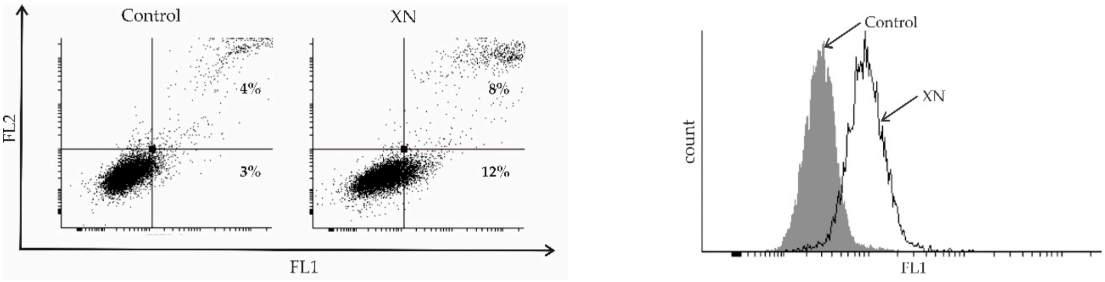

(**a**) (**b**)

Figure S28. Induction of apoptotic cell death by XN in B16F10 melanoma cell culture determined using Ann/PI (**a**) and ApoStat (**b**). All performed after 48 h of treatment with IC_{50} dose of XN and subsequently analyzed by flow cytometry. Dot plots and histograms are representative ones selected from three repeated experiments.

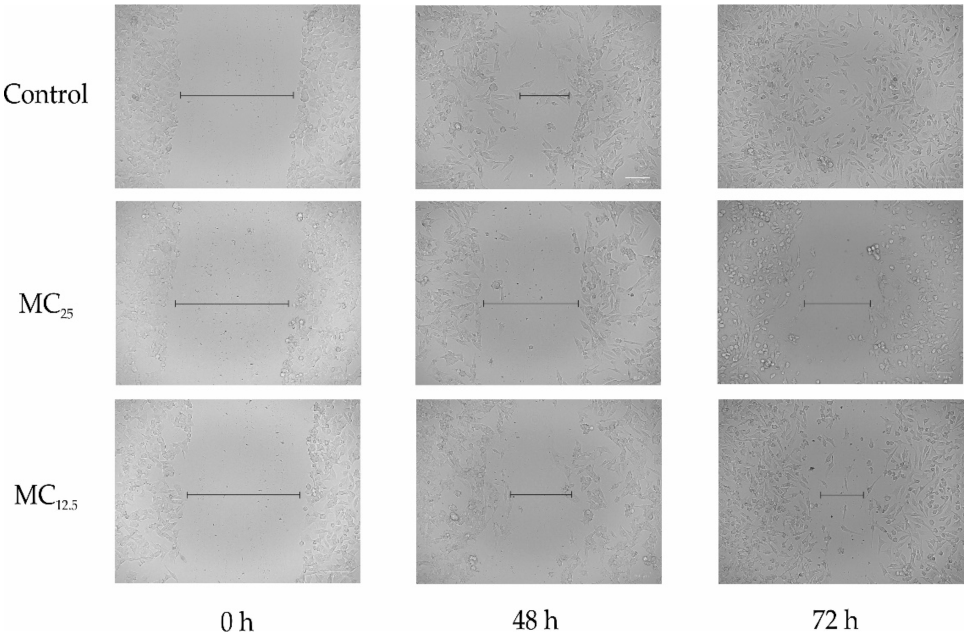

Figure S29. Suppression of B16F10 melanoma cell motility treated with $MC_{12.5}$ and MC_{25} doses of SBA-15|XN3 determined by wound healing assay. The cell cultures were observed under a microscope and digitally photographed at 3 different time points (0, 48 and 72 h).
